# LLM services in the management of social communications

**DOI:** 10.3389/frai.2025.1474017

**Published:** 2025-03-13

**Authors:** Yuriy Dyachenko, Oleksandra Humenna, Oleg Soloviov, Inna Skarga-Bandurova, Nayden Nenkov

**Affiliations:** ^1^Kyiv School of Economics, Kyiv, Ukraine; ^2^Institute of Education Content Modernization, Kyiv, Ukraine; ^3^Kremenchuk Mykhailo Ostrohradskyi National University, Kremenchuk, Ukraine; ^4^Oxford Brookes University, Oxford, United Kingdom; ^5^Konstantin Preslavsky University of Shumen, Shumen, Bulgaria

**Keywords:** dual-process theory, management of social communications, LLM services, behavioral economics, decision-making

## Abstract

This paper proposes enhancing social communication management with a behavioral economics approach through artificial intelligence instruments. The research aims to explore the influence of social communication on citizens’ behavior using large language model services and assess its effectiveness. The paper builds on Daniel Kahneman’s dual-process theory, highlighting the intuitive system (System 1) and the rational system (System 2) in decision-making. The author introduces a third system, System 3, representing rooted in identity socially conditioned behavior influenced by societal norms and self-awareness. On this theoretical basis, the paper emphasizes automating communication management through large language model services, freeing up citizens’ potential for self-determination and self-organization. By leveraging these services, messages can be crafted to support social transformation while respecting historical, cultural, and political contexts. Based on the preconditions and restrictions described above, we use GPT-4 model to generate messages based on these narratives. The experiment will use an observational study design with virtual persons. To compare the impact of original and modified messages according to the addressee’s mentality, we used the Claude 3.5 Sonnet model. We can see that the potential activity of respondents after perceiving the changed message does not change much, and the original message is perceived. Modifying messages by LLM services crafted to support social transformation while respecting historical, cultural, and political contexts cause attitudes to become substantially more negative (2.5 units downward shift in median); the intentions showed a slight positive increase (0.2 units upward change in median).

## 1 Introduction

This paper proposes enhancing social communication management with a behavioral economics approach through artificial intelligence instruments.

Many studies indicate that social communications undergo significant changes under the influence of digital technologies and social media (Halich et al., 2023; Ming and Salman, 2023). Social media increasingly play a crucial role in cultural and social life, shaping public opinion and stimulating discussions on important societal issues. Social communications also impact the development of intellectual processes in group situations, promoting the formation of “collective intelligence.” This occurs through adaptive communication networks that can change and restructure according to context, enhancing the efficiency of group decisions. Scientists note that such networks allow groups to exchange information better and compare different viewpoints, promoting optimal information flow and improving the quality of decisions made.

Social communications have dramatically transformed in the digital age, with social media platforms increasingly shaping public opinion, cultural discourse, and collective decision-making. These digital networks have created new “collective intelligence” forms through adaptive communication systems that allow groups to exchange information, compare perspectives, and make decisions more efficiently. However, this evolution in social communications presents opportunities and challenges for effectively managing public discourse and understanding its influence on citizen behavior.

The complexity of human decision-making in this social communications landscape can be understood through Daniel Kahneman’s dual-process theory, which describes two cognitive systems: the fast, intuitive System 1 and the slower, rational System 2. Building on this framework, the research proposes a third system – System 3 – which accounts for socially conditioned behavior shaped by cultural norms, identity, and collective values. This triadic model provides a more comprehensive framework for understanding how individuals make decisions within their social and cultural context, particularly in digital environments where social influence is increasingly pervasive.

The research proposes leveraging behavioral economics principles in conjunction with large language model (LLM) services to address the challenges of managing social communications in this complex landscape. This innovative approach aims to enhance the effectiveness of social communication management by accounting for all three cognitive systems – intuitive responses, rational deliberation, and socially conditioned behavior. By incorporating these advanced AI tools while considering the multifaceted nature of human decision-making, the research seeks to develop more nuanced and effective strategies for understanding and influencing citizen behavior through social communications.

Daniel Kahneman’s dual-process theory presents a fascinating framework for understanding human decision-making through two distinct cognitive systems: System 1 and System 2 (Kahneman, 2011). This model illuminates the dynamic interplay between intuition and rationality in our cognitive processes, impacting everything from mundane daily choices to critical life decisions.

System 1 operates automatically and quickly, with little or no effort and no sense of voluntary control. This system is often referred to as the intuitive system because it involves immediate, gut-response decision-making that does not require conscious thought. The operations of System 1 are typically fast, automatic, unconscious, and rely on heuristic patterns.

Conversely, System 2, the rational system, involves more deliberate, effortful, and conscious decision-making processes. It allocates attention to effortful mental activities that demand it, including complex computations and formulating reasoned arguments. System 2 is slower and more methodical, often engaging in a critical evaluation of the outcomes generated by System 1. This system is typically activated when a person needs to focus on a task that requires logical reasoning or when making decisions that require careful consideration, such as calculating a math problem or deciding on a moral dilemma.

The interplay between these two systems is crucial for understanding human behavior. System 1’s automatic operations can sometimes lead to biases and errors in judgment due to its reliance on associative memory and heuristic thinking. However, it is often efficient and effective for routine decision-making.

This dual-process approach to decision-making suggests that while our behavior may initially be guided by System 1’s fast and automatic responses, it is often overseen and corrected by System 2’s reflective capabilities. System 2 ensures that our actions align with broader cognitive evaluations and moral judgments. It acts as a monitor and a control mechanism that checks and occasionally corrects the impressions and decisions suggested by System 1.

The proposed work builds on seminal work in dual-process theory – for example, Kahneman’s (2011) distinction between fast, intuitive (System 1) and slow, deliberate (System 2) decision-making – and extends this framework by introducing a third system (System 3) that captures the influence of socially rooted, identity-driven behavior. This extension is motivated by critiques that binary models can be overly reductive when applied to complex social communication (e.g., Evans and Stanovich, 2013). Recent reviews have proposed integrating insights from embodied and predictive processing into dual-process models, arguing that System 1 processes are automatic and shaped by embodied social experiences (Bellini-Leite, 2022).

Comparative studies in behavioral economics have applied dual-process and nudging theories to practical interventions. For instance, randomized controlled trials have shown that well-designed nudges can reliably change health or safety behaviors by altering environmental cues. At the same time, emerging research on AI in behavioral economics has demonstrated that LLMs can be harnessed to automate persuasive communication. Rahwan and colleagues have conceptualized AI systems as social actors influencing trust and cooperation through human-like interaction patterns (Rahwan et al., 2019). Some perspectives, such as those articulated by Camerer on the interaction of AI with human decision-making, further support the promise of these technologies in complex social settings (Camerer, 2018).

The synthesis of these varied approaches suggests that while traditional social communication management has long relied on human-mediated control to safeguard authenticity and self-determination, recent advances in AI offer promising avenues for scalable, personalized interventions. By introducing System 3, the current proposal seeks to integrate dual-process insights with social identity and norm-based theories, accounting for culturally and historically situated communication practices. Nevertheless, several challenges remain. In particular, the operationalization of System 3 is still under debate, and conflicts persist between the conventional wisdom favoring autonomous citizen engagement and the risks of algorithmic manipulation. The comparative Table 1 summarizes the theoretical underpinnings, methodologies, and limitations of related works in this emerging field.

**Table 1:** Comparative table of related works.

Study/approach	Theoretical framework	Methodology	Key Findings	Limitations
Kahneman’s dual-process theory	Fast (System 1) vs. slow (System 2) decision-making (Kahneman, 2011); extended by Evans and Stanovich (2013)	Reviews of experimental studies on judgment and decision-making	Established robust evidence for two distinct cognitive processes	Criticized for oversimplifying the nuanced effects of social identity and contextual influences
Embodied and predictive extensions	Integration of embodied cognition with dual-process models (Bellini-Leite, 2022)	Meta-analyses and conceptual reviews using neuroimaging and behavioral experiments	Suggest that automatic responses (System 1) are shaped by embodied experiences, urging the addition of further processing layers	Ongoing debate regarding the operational definition and empirical separability of the proposed third system (System 3)
Nudging interventions in behavioral economics	Libertarian paternalism and choice architecture (Thaler and Sunstein, 2008)	Randomized controlled trials (RCTs) in health, finance, and safety interventions	Demonstrated that subtle changes in choice architecture can effectively shift behavior	Effectiveness varies with context and individual differences; ethical concerns regarding manipulation
AI empowerment in behavioral sciences	Integration of behavioral economics with AI tools (Thaler and Sunstein, 2008)	Field experiments and case studies using LLMs for message generation	Personalized AI interventions can enhance decision-making efficiency and support social communication	Challenges remain in scaling interventions and mitigating algorithmic biases; often reliant on proprietary platforms
Machine behavior and social actor models	Conceptualizing AI systems as social actors using computational social science frameworks (Rahwan et al., 2019)	Mixed-method approaches, including observational studies and experimental designs	AI systems display human-like interaction patterns that can influence trust and cooperative behavior	Debate continues whether AI can fully replicate human social behavior; ethical and transparency issues persist
Proposed approach (enhancing social communication management)	Extension of dual-process theory by adding System 3 to account for socially conditioned, identity-driven behavior	Observational study design with virtual persons using LLMs for automated message generation	Suggests that automated communication management can free citizens’ potential for self-determination while enabling social transformation	Novelty of System 3 definition; observational design limits causal inference; potential conflicts with traditional views on citizen autonomy

## 2 Materials and methods

### 2.1 Enhancing the dual-process theory of decision-making

Building on Daniel Kahneman’s dual-process theory of human cognition, which identifies System 1 (intuitive) and System 2 (rational) as key frameworks in decision-making, it is beneficial to consider introducing a third system, System 3, to capture a broader spectrum of human behavior foundations. This proposed System 3 would represent socially conditioned behavior influenced by societal norms and self-awareness, encapsulating the intricate ways in which our decisions are shaped not only by subconscious and rational factors but also by our sociocultural environment.

Proposed System 3 reflects the influencing of social conditioning, identity, and societal norms on behavior and decision-making, which were investigated within the framework of Social Identity Theory (Tajfel and Turner, 1979), explains how people’s sense of self is derived from group memberships and social categories, which then influence behavior and decision-making. From this point of view, a person who strongly identifies as environmentally conscious may automatically choose eco-friendly products without engaging in the cost–benefit analysis typical of System 2 thinking, yet this is not quite the automatic, instinctive processing of System 1 either.

Within the Cultural-Historical Activity Theory (Vygotsky, 1978; Leontiev, 1978), cultural and historical contexts shape human behavior and cognition through internalized social practices. From this point of view, for example, the way people queue in different cultures – British people tend to form orderly lines automatically, while other cultures might have different spatial arrangements for waiting that are neither purely instinctive (System 1) nor calculated (System 2).

Habitus Theory (Bourdieu, 1977) explains how social conditioning becomes embodied in individuals, creating durable dispositions that guide behavior without conscious calculation. For example, class-based differences in food preferences or cultural tastes that feel “natural” to individuals but are socially conditioned.

Socially conditioned behaviors influenced by identity and norms are very real. System 3 can integrate its aspects into the decision-making framework to consider the impact of social and cultural influences on cognition and behavior.

System 3 may reflect a more reasoned decision-making process in which inputs from sensory experiences are integrated and analyzed based on an individual’s values and goals. Furthermore, System 3’s sensitivity to feedback loops based on human values implies that our reflective processes are logical and influenced by our personal and societal norms.

System 3 can be understood as a very slowly changing socially conditioned system rooted in identity that plays a pivotal role in behaviors driven by societal norms and expectations. It involves an awareness of societal values and the reflexive ability to adjust individual behavior following these values, which can be described as “decent” or socially responsible behavior. For instance, when individuals decide to follow recycling guidelines or adhere to public health advisories, they are likely influenced by System 3, which mediates personal actions with a collective ethical framework.

This introduction provides an opportunity to integrate the cognitive influences of Systems 1 and 2 with the external social context, creating a triadic model of human cognition in which behavior is also a function of social conditioning and identity. System 3 is slower in its evolution, reflecting the gradual changes in cultural norms and societal values over time. It provides a feedback mechanism incorporating societal approval or disapproval into personal decision-making processes, influencing long-term behavioral patterns and ethical considerations.

Moreover, System 3 enriches our understanding of identity as it interacts with subconscious instincts (System 1) and rational considerations (System 2). Identity in this context is not merely about personal introspection or rational self-assessment but also involves how individuals see themselves in the social mirror. This aspect of self-awareness is shaped by cultural, social, and historical forces, and it affects how individuals conform to or rebel against societal expectations.

Incorporating System 3 into the dual-process framework may allow for a more nuanced understanding of human behavior, including the influence of socially determined norms. This triadic model explains individual decision-making and enhances our comprehension of group behaviors and societal trends. It accounts for the complexity of decisions not entirely based on instinct or reason but also deeply embedded in a social matrix that dictates what is considered appropriate, responsible, or ethical.

Thus, for a comprehensive analysis of human behavior, it is essential to consider all three systems: the subconscious instincts governed by System 1, the rational deliberations of System 2, and the socially conditioned behaviors of System 3. Each system interacts with the others, creating a dynamic interplay that profoundly shapes individual and collective behavior. By acknowledging the role of societal norms and identity (System 3), we gain a fuller picture of the factors that drive human actions and the societal frameworks that guide them.

### 2.2 Application of the enhanced dual-process theory of decision-making for social communication management

Social communication management represents a critical dimension of understanding human interactions within various contexts, particularly considering the extended framework of human cognition involving Systems 1, 2, and 3 – where System 3 specifically deals with socially conditioned behavior.

System 1, the intuitive system, is implicit in social communication by facilitating quick, automatic responses to social stimuli. This system allows individuals to respond to social cues quickly and efficiently, crucial in dynamic social interactions.

System 2, the rational system, introduces a more deliberate layer to social communication. It governs how we process complex linguistic constructs, interpret semantic ambiguities, and engage in thoughtful discourse.

System 3, based on identity, as introduced, encompasses the socially conditioned behaviors heavily influenced by societal norms and cultural expectations. This system is pivotal in managing social communication because it guides individuals on appropriate social behaviors and communication ethics (Maia, 2014). It involves a higher level of self-awareness and reflection (Dyachenko et al., 2018), allowing individuals to adjust their communicative practices to align with social norms and values.

Effective social communication management, therefore, requires an interplay of all three systems. The intuitive reactions from System 1 need to be balanced with the thoughtful analysis of System 2 and the ethical considerations of System 3. This balance is crucial in diverse social contexts, such as multicultural interactions, where miscommunications can arise from varying social norms and expectations.

However, managing this balance presents challenges. Over-reliance on System 1 can lead to premature judgments or cultural faux pas, whereas excessive deliberation in System 2 can result in communication that is perceived as insincere or overly calculated. Moreover, System 3’s slow adaptability might lag rapidly changing societal norms, leading to outdated or inappropriate responses in new social milieus.

The ability to comprehend and engage with a message is influenced by intelligence, available time, knowledge level, environmental distractions, and message repetition frequency. People are more likely to respond to messages that align with their existing knowledge (Ming and Salman, 2023).

Therefore, social communication management involves understanding and integrating cognitive processes across all three systems. By fostering awareness of these systems and their impact on communication, individuals and organizations can enhance their interactions within and across cultural boundaries, leading to more effective and harmonious social relations. This approach improves interpersonal communications and equips society to better navigate the challenges of an increasingly interconnected world.

### 2.3 Artificial intelligence instruments for communication management

Integrating AI instruments (Zerfass et al., 2020), particularly LLMs, into communication management (Seidenglanz and Baier, 2023) represents a significant advancement in technology and social interaction. We can use AI-driven tools to enhance communication strategies by utilizing the cognitive systems framework: System 1 (intuitive), System 2 (rational), and System 3 (socially conditioned), as discussed previously.

LLMs like GPT (Generative Pre-trained Transformer) have revolutionized the field of natural language processing (NLP) by enabling machines to understand and generate human-like text. These models operate at the intersection of all three cognitive systems. They mimic System 1 by rapidly generating responses based on patterns learned from vast datasets, facilitating quick and intuitive communication. In System 2, LLMs assist in processing complex information and producing reasoned, coherent outputs necessary for detailed explanations or problem-solving tasks in communications. Finally, through learned social and cultural nuances, LLMs reflect aspects of System 3 by adhering to societal norms and ethical guidelines in their outputs. This is crucial for maintaining decency and appropriateness in communication.

In content creation, AI tools are adept at generating written content for blogs, reports, and marketing materials. They assist in crafting messages that are not only grammatically correct and stylistically appropriate but also tailored to the cultural context of the target audience, showcasing their alignment with System 3.

In social media management, LLMs analyze and generate content for social media platforms, managing posts and interactions to conform to social norms. They can moderate discussions, filter inappropriate content, and maintain a brand’s voice across channels, demonstrating an advanced application of System 3 in communication.

While LLMs offer substantial benefits, their application in communication management is not without challenges. One major concern is the potential for these models to propagate biases in their training data, which can lead to inappropriate or harmful communication outputs. This issue ties directly into the ethical considerations of System 3, where societal norms are paramount – ensuring that LLM outputs are unbiased and representative requires continuous monitoring and updating of AI models to reflect evolving societal values.

Another challenge is the risk of dependency on automated systems, which might degrade human cognitive abilities in Systems 2 and 3. Relying too heavily on AI for communication tasks might diminish individuals’ ability to engage deeply with complex information or navigate social nuances independently.

Thus, applying LLMs in communication management demonstrates a powerful synergy between AI technology and human cognitive frameworks. By enhancing intuitive, rational, and socially conditioned communication processes, LLMs improve the efficiency and effectiveness of communication strategies and present new opportunities and challenges in the digital age. As these tools become more integrated into societal frameworks, responsibly managing their development and usage will be crucial to maximizing their benefits while mitigating risks. This will ensure that AI-enhanced communication supports broader equity goals, ethical interaction, and cultural sensitivity.

### 2.4 Application of LLM services for communication management to free up citizens’ potential for democratic transformations

Integrating LLMs in communication management can significantly influence democratic processes by freeing up citizens’ potential for participation and engagement. LLM services can enhance democratic transformations by facilitating informed discourse and engagement, aligned with the cognitive systems framework: System 1 (intuitive), System 2 (rational), and System 3 (socially conditioned).

Through their capacity to process and generate vast amounts of information quickly, LLMs can democratize access to complex data and policy discussions. By simplifying intricate government documents, legal texts, and policy debates into more accessible language, LLMs can empower citizens by making information more understandable and engaging. This aligns with System 1, providing intuitive grasps of complex subjects without overwhelming cognitive load, thus encouraging broader public participation in democratic discourse.

Regarding System 2 operations, LLMs can contribute to more rational and informed public discussions by providing data-driven insights and balanced perspectives. For instance, during election periods, LLMs can analyze candidates’ proposals, offering unbiased summaries and comparisons based on historical data and policy analysis. This rational, evidence-based approach to communication helps counter misinformation and promote critical thinking among the electorate, which is essential for informed voting and civic participation.

Reflecting System 3, LLMs can be pivotal in maintaining and promoting democratic norms and social cohesion. By moderating online forums and social media platforms, LLMs can help enforce community guidelines that discourage hate speech and encourage respectful discourse. Additionally, LLMs can facilitate cross-cultural and inter-community dialogues by translating content and mediating discussions, fostering inclusivity and mutual respect among diverse groups.

However, deploying LLMs in democratic contexts must be navigated carefully to avoid potential pitfalls. The risk of perpetuating existing biases, manipulating opinions, or infringing on privacy remains significant. Ensuring that LLMs operate transparently and ethically is paramount to maintaining trust in democratic institutions and processes. Continuous monitoring and adaptive frameworks are necessary to align LLM outputs with evolving societal values and democratic principles.

Moreover, the dependency on technology for democratic engagement could lead to disparities in participation among different demographic groups, especially those with limited access to digital technologies or those less technologically literate. Addressing these disparities is crucial to ensure that the benefits of LLMs in democratic transformations are equitably distributed.

Applying LLMs in communication management holds considerable potential to enhance democratic transformations by making information more accessible, supporting rational discourse, and reinforcing social norms conducive to democracy. By effectively integrating these systems into democratic processes, LLMs can help free up citizens’ potential for active and informed participation. However, managing these technologies to ensure they uphold democratic values without compromising ethical standards or social equity will be critical in realizing their full potential in supporting democratic transformations. This approach not only enhances the immediate efficiency of democratic engagements but also contributes to the long-term resilience and inclusivity of democratic institutions.

Modification messages and receiving feedback through simulated virtual personalities through LLM services are two ways to manage the impact on audience attitudes and behavioral intentions. LLMs, such as OpenAI’s GPT series, can generate human-like text, enabling the tailoring of messages to specific audiences. This customization can enhance message effectiveness by aligning content with audience preferences and expectations. However, the ethical implications of such modifications, including concerns about authenticity and manipulation, warrant careful consideration.

Recent studies have explored the impact of virtual personalities in LLM-driven communications. Hu and Collier (2024) investigated how incorporating persona variables – demographic, social, and behavioral factors – affects LLMs’ ability to simulate diverse perspectives. Their findings suggest that while persona – integration offers modest improvements in simulation accuracy, the overall effect size is limited, indicating that persona variables account for less than 10% of the variance in human annotations.

The effectiveness of personality-adaptive chatbots has also been systematically reviewed; Ait Baha et al. (2023) analyzed 66 studies focused on chatbots that adapt to users’ personalities, highlighting various deep learning approaches for personality recognition and adaptation. The review emphasizes aligning chatbot responses with user personalities to enhance engagement and satisfaction. However, challenges remain in accurately recognizing and adapting to diverse personality traits.

Furthermore, the ability of LLMs to emulate human personalities has been examined. Li et al. (2024) demonstrated that supervised fine-tuning can more effectively shape LLM personalities than prompt-based approaches, resulting in higher validity and consistency in personality emulation.

**Figure 1:**
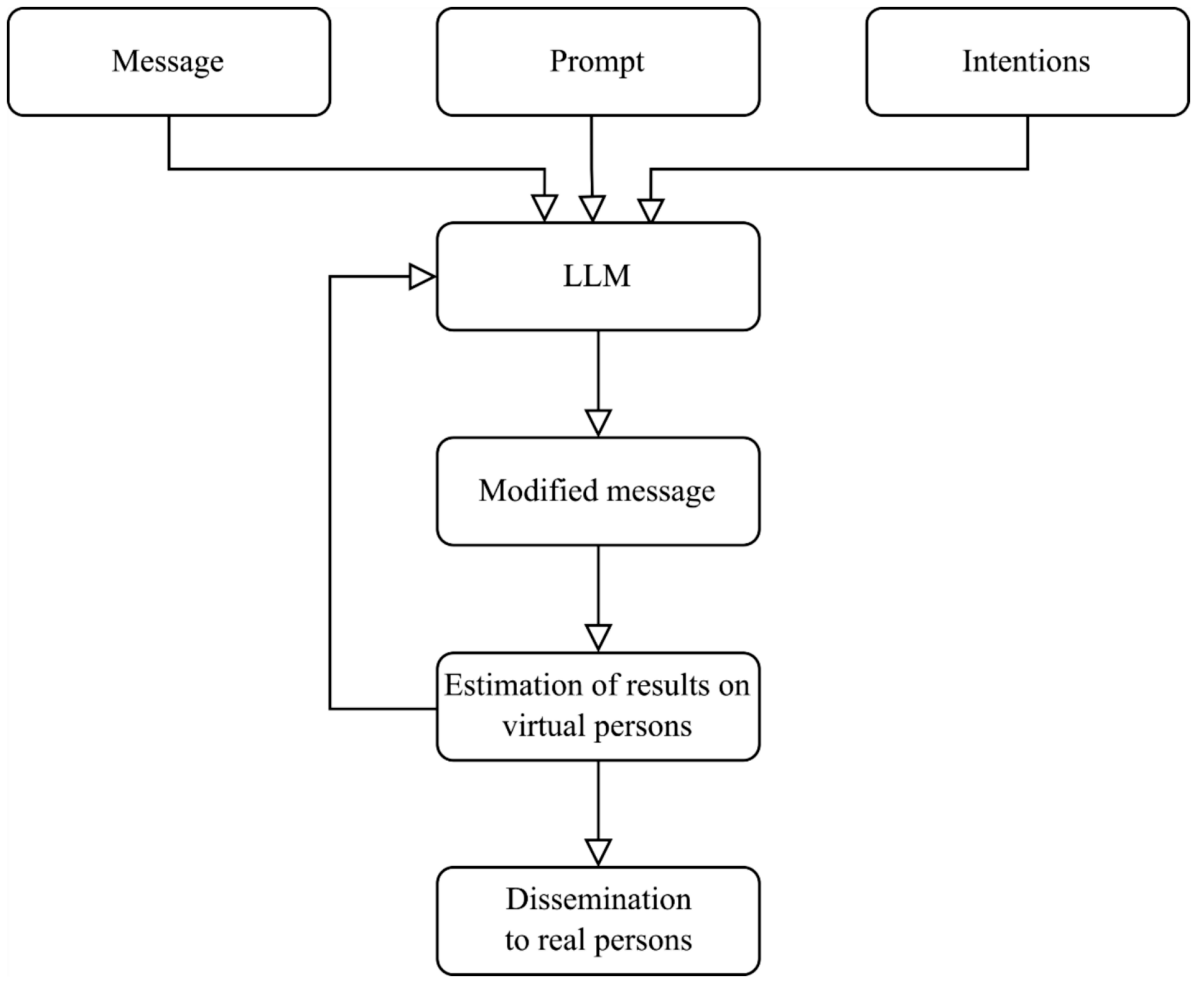
Scheme of proposed modification of messages by LLM.

In this work, we propose a low-requirements technique using LLM prompts to modify messages targeting specific behaviors or attitudes by tone, framing, and content. We used GPT-4 model from OpenAI to generate messages and Claude 3.5 Sonnet model from Anthropic to access an LLM and compare the impact of original and modified messages. This technique explores different aspects of identity and worldview alignment. The effectiveness will be estimated by the impact on the behavior of virtual personalities created through the LLM service (Figure 1).

## 3 Results

### 3.1 Qualitative and quantitative analysis of the effectiveness of the impact of artificial intelligence instruments on social communication

Practically, we emphasize automating communication management through LLM services, freeing up citizens’ potential for self-determination and self-organization. By leveraging these services, messages can be crafted to support social transformation while respecting historical, cultural, and political contexts.

We can design narratives in support of the transformation of the country with a deep understanding of the historical, cultural, and political contexts. The narratives should be respectful, informed, and considerate of the diverse perspectives within the country. We can propose the following narrative topics that could be explored, considering the need for sensitivity and respect for historical diversity, decentralization of power, economic opportunities, cultural renaissance, peace and cooperation, environmental management, and global integration.

Based on the preconditions and restrictions described above, we used GPT-4 model from OpenAI to generate messages on these narratives.

Designing an experiment to assess the effectiveness of LLM-generated messages without direct feedback from participants requires indirect evaluation methods. Such an experiment would rely on observable behaviors and data analysis techniques to infer the effectiveness of communication based on behavioral economics principles and cognitive responses. Here’s a proposed experiment design overview.

The objective is to evaluate the effectiveness of messages generated by LLMs in terms of identity resonance, worldview alignment, and loyalty induction using observable behaviors of virtual persons.

Using virtual personalities through LLM services for survey simulations gives researchers a powerful tool to explore diverse response patterns in a controlled environment. By crafting and deploying distinct personas with varying backgrounds, attitudes, and preferences, researchers can simulate various respondent types without relying exclusively on real-world participants. This approach broadens the scope of data collection and addresses ethical and logistical concerns, as it minimizes the need to involve human subjects in repetitive or sensitive questioning.

The robustness of this approach arises from the ability to test and validate findings against multiple simulated perspectives. Instead of relying on a single, uniform data source, researchers can compare and contrast results across various personas, each serving as an independent test case. This triangulation of evidence bolsters the credibility of any conclusions drawn, ensuring that the observed patterns are not merely artifacts of one particular group or sample. Consequently, using virtual personalities in LLM-based survey simulations offers a scalable, ethically sound, and methodologically robust means of generating and testing hypotheses in various research fields.

We propose the following experimental design:

1. Message creation. We use an LLM to generate multiple versions of messages targeting specific behaviors or attitudes. These messages should vary systematically in tone, framing, and content to explore different aspects of identity and worldview alignment. Alongside LLM-generated messages, we create control messages using standard communication practices.
2. Behavioral modelling. To take engagement metrics, we can track user interactions with each message, such as likes, shares, comments, and time spent on message pages.
3. Data analysis. Comparing engagement and behavior metrics between messages to determine which versions most effectively influence behavior suggests higher effectiveness regarding identity resonance and worldview alignment.

As the key variables, we define the following:

1. Independent variable – the message type (LLM-generated vs. control).
2. Dependent variable – user engagement.

To provide data privacy, we ensure all data collection complies with privacy laws and platform policies, using only anonymized, aggregated data for analysis. While direct feedback is not collected, ensure users know general data usage policies on platforms.

As a limitation, we see:

1. Interpretation bias that, without direct feedback, requires consideration that must be made about why users engage differently with various messages.
2. External factors like current events or social trends could affect user behavior independently of the messages.

This experiment design leverages indirect measures to assess the impact of LLM-generated messages on user behavior, providing insights into their effectiveness in communication management. By analyzing engagement in response to different message strategies, one can infer how well messages resonate with user identities and worldviews and how effectively they induce loyalty without direct user feedback.

Concerning difficulties in disseminating and reviewing feedback on messages, we propose using virtual persons.

We used the Claude 3.5 Sonnet model from Anthropic to access an LLM and compare the impact of original and modified messages according to the addressee’s mentality.

Argyle et al. (2023) shows that the GPT-family language model is “both fine-grained and demographically correlated, meaning that proper conditioning will cause it to emulate response distributions from various human subgroups accurately. It is nuanced, multifaceted, and reflects the complex interplay between ideas, attitudes, and socio-cultural context that characterize human attitudes”.

But in Dillion et al. (2023) outlined caveats of using AI as a participant: “LLMs may be most useful as participants when studying specific topics, when using specific tasks, at specific research stages, and when simulating specific samples”.

We created 10 virtual personalities and set the task of modeling their response to the original and modified information messages.

We evaluated the responses on two scales:

1. Attitude to the message on a scale from −3 “extremely negative” to +3 “extremely negative.”
2. Intention to act due to the influence of the information received on a scale from 0, “I am not going to do anything at all,” to 3, “I will definitely take certain actions.”

**Figure 2:**
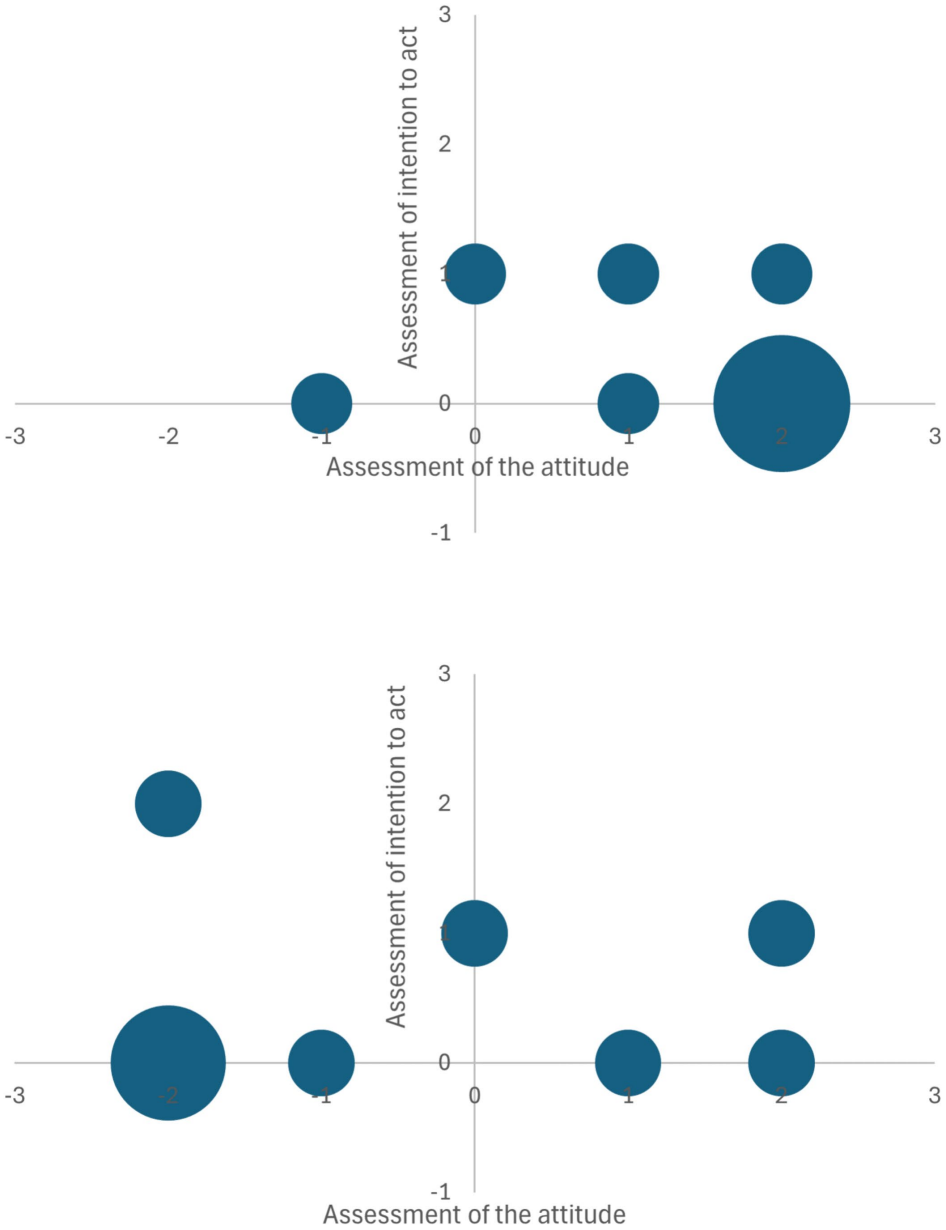
Graphical interpretation of the estimates of virtual respondents’ response to the original (top) and modified information messages (bottom).

The graphical interpretation of the estimates of virtual respondents’ answers is shown in Figure 2.

## 4 Discussion

We can see that the potential activity of respondents after perceiving the changed message does not change much, and the original message is perceived.

As a result of changing the message by LLM service, we got the following results:

1. Respondents’ attitudes became substantially more negative (2.5 units downward shift in median).
2. The intentions showed a slight positive increase (0.2 units upward shift in median).
3. There is a decoupling between attitudes and intentions in the modified message condition.

Generally, messages crafted to support social transformation while respecting historical, cultural, and political contexts tend to be more pessimistic about events and significantly impact the intention to act.

The growing willingness to act under the influence of modified messages can free up citizens’ potential for self-determination and self-organization.

However, in the future, we must consider ways to reduce the decoupling between attitudes and intentions in the modified message condition.

One of the primary difficulties in using virtual personalities for survey simulations lies in ensuring the authenticity and representativeness of the generated responses. Although LLMs can capture various conversational styles and viewpoints, these may not perfectly mirror the diversity of real human populations. In some cases, the pretraining data for LLMs might be skewed toward specific demographics or cultural contexts, limiting the models’ capacity to simulate particular population segments reliably. Overcoming this hurdle involves curating high-quality, balanced datasets and continuously refining the underlying models to incorporate a broad spectrum of cultural, geographical, and socio-economic factors. Researchers may also need to apply domain-specific fine-tuning so that the virtual personalities accurately reflect the nuances of the populations they aim to represent.

Another challenge arises in managing and mitigating the risk of unintended biases or inconsistencies introduced by virtual personas. Since LLMs can inadvertently reproduce prejudices embedded in their training data, certain personality constructs may display biased or conflicting views across different topics. To address this issue, researchers can employ systematic bias-detection protocols – such as leveraging third-party tools or custom scripts to analyze generated text for indicators of stereotyping or discrimination. Implementing iterative feedback loops, wherein researchers test simulated survey results against smaller-scale real-world samples, can help ensure that the virtual personalities remain as bias-free and consistent as possible.

While these advancements offer promising avenues for enhancing communication through LLMs and virtual personalities, it is crucial to address ethical considerations. The potential for manipulation and the authenticity of AI-generated content pose significant challenges. Ongoing research is essential to develop guidelines and frameworks that ensure the responsible use of LLMs in modifying messages and simulating virtual personalities, thereby safeguarding against ethical pitfalls and promoting trust in AI-mediated communications.

Due to significant ethical challenges, particularly concerning biases and manipulation in integrating AI into social communication management, for instance, gender and racial biases, reinforcing stereotypes, and marginalizing certain groups (Rallabandi et al., 2023), several strategies can mitigate these ethical concerns. We must incorporate diverse and representative data during the training phase to minimize inherent biases encompassing a wide range of demographics and perspectives, which can lead to more equitable AI outcomes. Another effective strategy is the interdisciplinarity through establishing teams in AI development with ethicists, sociologists, and domain experts alongside engineers, organizations who can better anticipate and address ethical dilemmas related to AI deployment in social communication that fosters a more holistic understanding of potential impacts and promotes the creation of systems that align with societal values. Regular audits and assessments of AI systems by conducting periodic evaluations to detect and rectify biases and ensure that AI tools remain fair and effective over time are also essential. Adhering to established ethical guidelines and frameworks, such as The Toronto Declaration (2018) is vital in guiding the responsible use of AI in social communication management to emphasize the protection of human rights in machine learning systems, advocating for accountability and the mitigation of discrimination.

## 5 Conclusion

The research reveals insights into leveraging large language model (LLM) services for social communication management by introducing an innovative triadic cognitive framework that extends Kahneman’s dual-process theory. By proposing System 3 – a socially conditioned behavioral system rooted in identity and cultural norms – the study provides a more nuanced understanding of human decision-making processes. Integrating AI technologies with this enhanced cognitive model demonstrates the potential for more sophisticated communication strategies that respect historical, cultural, and political contexts while facilitating democratic transformations.

Experimental results using virtual personalities highlight the promise and challenges of AI-driven communication management. While LLM-modified messages showed a significant negative shift in attitudes (2.5 units downward), they paradoxically generated a slight positive increase in behavioral intentions (0.2 units upward). This unexpected outcome underscores the complex interactions between message framing, cognitive systems, and potential behavioral responses. The research emphasizes the need for careful, ethically-guided approaches to AI-mediated communication that avoid manipulation while supporting informed civic engagement.

Looking forward, this research opens critical avenues for future exploration in AI-enhanced social communication. Key challenges remain in mitigating potential biases, ensuring authentic representation, and developing robust frameworks for responsible AI deployment. Interdisciplinary collaboration involving technologists, ethicists, sociologists, and communication experts will be crucial in refining these approaches. By continuing to develop nuanced, context-sensitive AI communication tools that respect individual and collective cognitive processes, we can potentially unlock new pathways for more inclusive, informed, and transformative social interactions.

## Data Availability

The raw data supporting the conclusions of this article will be made available by the authors, without undue reservation.
